# P-992. Impact of Ertapenem De-restriction on Hospital Length of Stay for ESBL-producing Enterobacterales Bloodstream Infections

**DOI:** 10.1093/ofid/ofaf695.1191

**Published:** 2026-01-11

**Authors:** Sarah B Green, Benjamin Albrecht, Sujit Suchindran, Lucy S Witt

**Affiliations:** Emory University Hospital, Atlanta, Georgia; Emory University Hospital, Atlanta, Georgia; Emory University School of Medicine, Atlanta, GA; Emory University, Atlanta, Georgia

## Abstract

**Background:**

Traditional antimicrobial stewardship initiatives aimed at reducing drug acquisition expenditures may fail to capture overall healthcare costs including increased hospital length of stay (LOS). Meropenem (MEM) had historically been utilized at our institution for treatment of extended-spectrum beta-lactamase producing Enterobacterales (ESBL-E) bloodstream infections (BSI) due to the high cost of ertapenem (ETP). Drugs requiring multiple daily infusions like MEM are associated with additional expenses for nurse time, intravenous tubing, admixture fluids, syringes and waste disposal compared to once-daily ETP and have also been associated with increased LOS. We aimed to quantify differences in LOS and other associated healthcare costs for patients with ESBL-E BSI treated with MEM versus ETP following de-restriction of ETP for Infectious Diseases providers in the inpatient setting.

**Figure:**
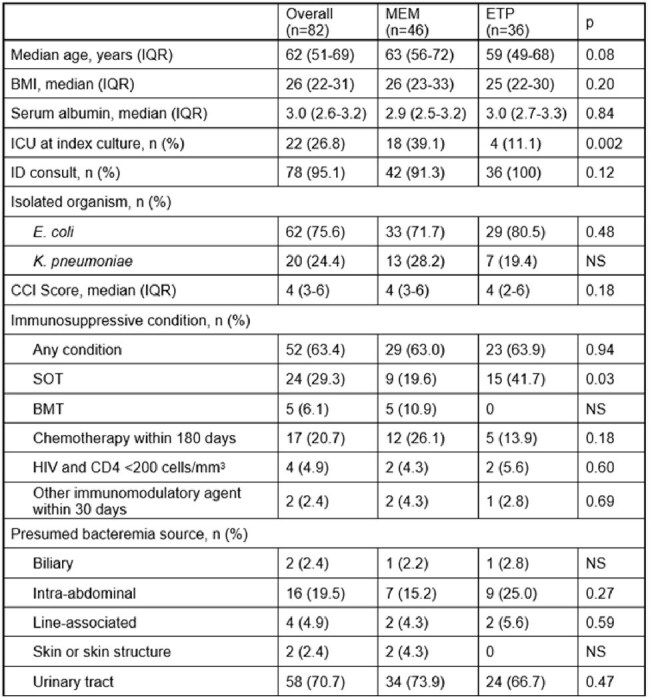
Baseline patient characteristics. Abbreviations: BMI = body mass index; BMT = bone marrow transplant within the previous 12 months; CCI = Charlson Comorbidity Index; ETP = ertapenem; HIV = human immunodeficiency virus; ICU = intensive care unit; ID = infectious diseases; IQR = interquartile range; MEM = meropenem; NS = non-significant; SOT = any history of solid organ transplant

**Figure:**
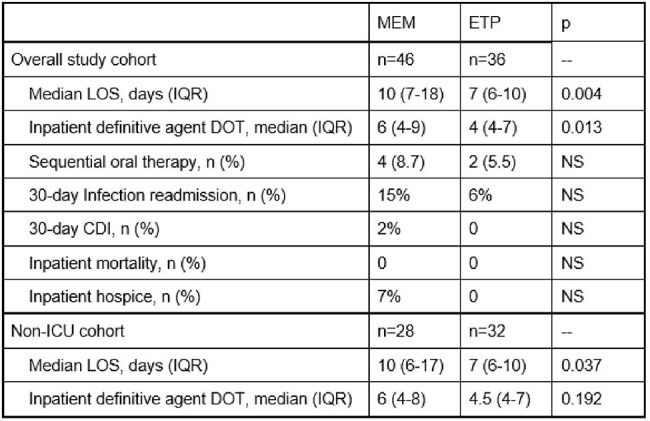
Length of stay, antimicrobial use, and patient outcomes. Abbreviations: CDI = C. difficile infection; DOT = days of therapy; ETP = ertapenem; ICU = intensive care unit; IQR = interquartile range; LOS = length of stay; MEM = meropenem; NS = non-significant

**Methods:**

This retrospective study included patients with ceftriaxone-resistant *E. coli* or *K. pneumoniae* BSIs between February 1, 2023 and March 31, 2025. Patients were included if they received at least 72 hours of therapy with ETP or MEM. Patients with polymicrobial bacteremia, those receiving combination therapy for > 72 hours, or infections without documented source control were excluded.

**Figure:**
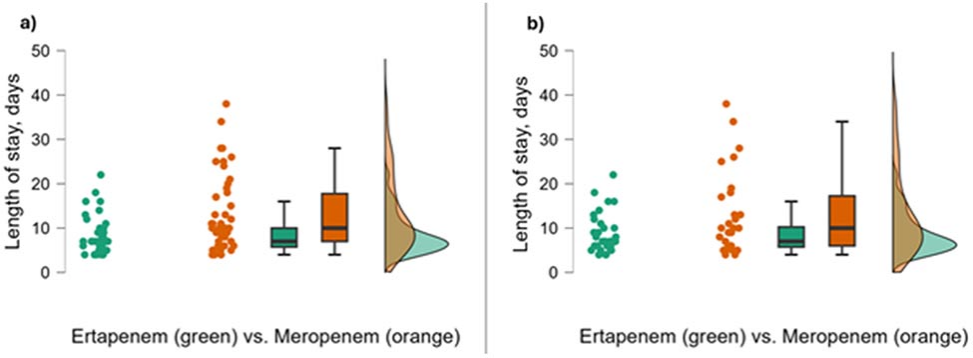
Raincloud plots of LOS for (a) overall and (b) non-ICU cohorts. a) LOS was significantly decreased in the ETP group for the overall study cohort (median 7 [IQR: 6-10] versus 10 days [IQR: 7-18], p <0.004). b) LOS was significantly decreased in the ETP group for the subset of patients not admitted to the ICU (median 7 [IQR: 6-10] versus 10 days [IQR: 6-17], p <0.037). Abbreviations: ETP = ertapenem; ICU = intensive care unit; IQR = interquartile range; LOS = length of stay; MEM = meropenem

**Figure:**
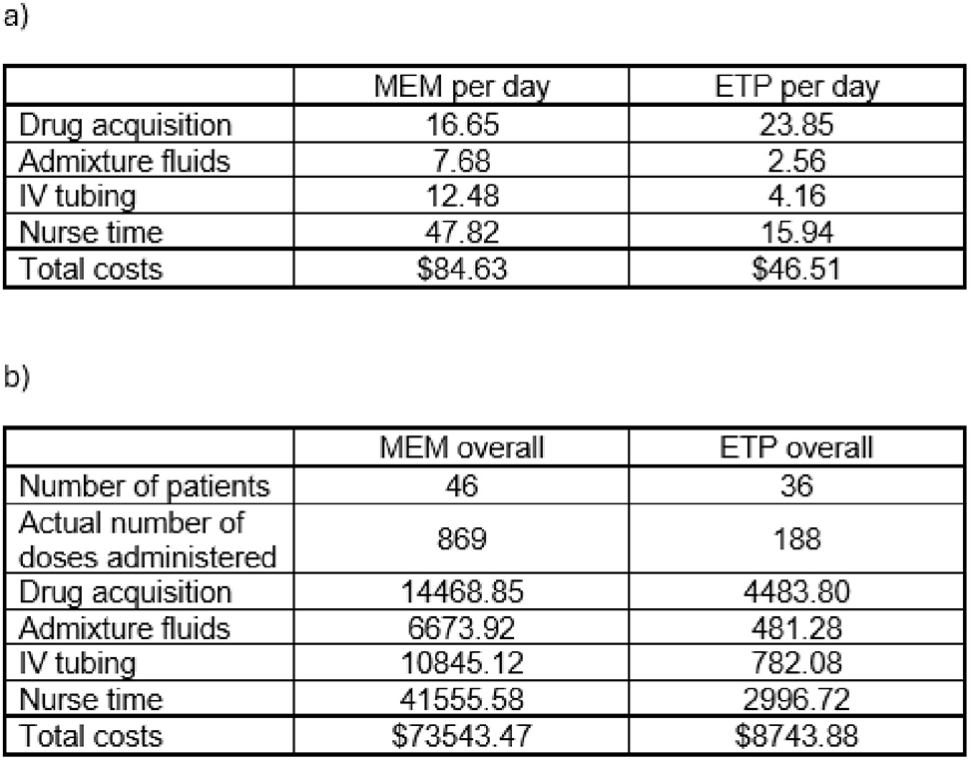
Associated costs of treatment, dollars. a) Associated costs per day of MEM and ETP administration for a patient with normal renal function and BMI <30; MEM is administered as 1 gram IV q8h over 3h and ETP 1 gram IV q24h over 30 minutes per institutional protocol. b) Total costs of MEM and ETP for all study patients based on dosing frequency and length of definitive therapy. Abbreviations: ETP = ertapenem; IV = intravenous; MEM = meropenem

**Results:**

Of the 161 blood cultures reviewed, 82 patients met criteria for study inclusion, 46 in the MEM group and 36 in the ETP group. Baseline characteristics were comparable in both groups, except for more patients in the MEM group admitted to the ICU at the time of index culture (Figure 1). More than 63% of patients included had an immunosuppressive condition. LOS was decreased in the ETP group compared to MEM in the overall study population (7 versus 10 days, p < 0.004) and in the subset of patients not admitted to the ICU (p < 0.037) (Figures 2 and 3). Associated costs of administration were also decreased (Figure 4).

**Conclusion:**

A change to de-restriction of inpatient ETP use for ESBL *E.coli* and *K. pneumoniae* BSI resulted in a significant decrease in patient LOS. Associated healthcare costs were also decreased despite the increased acquisition cost of ETP. Antimicrobial stewardship programs should look beyond traditional drug cost reduction initiatives to optimize overall healthcare savings and patient outcomes.

**Disclosures:**

Lucy S. Witt, MD, MPH, Merck & Co: Grant/Research Support

